# Optogenetic stimulation of the motor cortex alleviates neuropathic pain in rats of infraorbital nerve injury with/without CGRP knock-down

**DOI:** 10.1186/s10194-020-01174-7

**Published:** 2020-08-26

**Authors:** Jaisan Islam, Elina KC, Byeong Ho Oh, Soochong Kim, Sang-hwan Hyun, Young Seok Park

**Affiliations:** 1 Department of Neuroscience, College of Medicine, Chungbuk National University, Cheongju, South Korea; 2 Department of Neurosurgery, Chungbuk National University Hospital, Cheongju, South Korea; 3 ISCRM, Department of Veterinary Medicine, College of Veterinary Medicine, Chungbuk National University, Cheongju, South Korea; 4 Department of Neurosurgery, Chungbuk National University Hospital, College of Medicine, Chungbuk National University, 776, 1 Sunhwanro, Seowon-gu, Cheongju-Si, Chungbuk 28644 South Korea

**Keywords:** Optogenetics, Neuropathic pain, Trigeminal ganglion, α-CGRP, Thalamus, Motor cortex

## Abstract

**Background:**

Previous studies have reported that electrical stimulation of the motor cortex is effective in reducing trigeminal neuropathic pain; however, the effects of optical motor cortex stimulation remain unclear.

**Objective:**

The present study aimed to investigate whether optical stimulation of the primary motor cortex can modulate chronic neuropathic pain in rats with infraorbital nerve constriction injury.

**Methods:**

Animals were randomly divided into a trigeminal neuralgia group, a sham group, and a control group. Trigeminal neuropathic pain was generated via constriction of the infraorbital nerve and animals were treated via selective inhibition of calcitonin gene-related peptide in the trigeminal ganglion. We assessed alterations in behavioral responses in the pre-stimulation, stimulation, and post-stimulation conditions. In vivo extracellular recordings were obtained from the ventral posteromedial nucleus of the thalamus, and viral and α-CGRP expression were investigated in the primary motor cortex and trigeminal ganglion, respectively.

**Results:**

We found that optogenetic stimulation significantly improved pain behaviors in the trigeminal neuralgia animals and it provided more significant improvement with inhibited α-CGRP state than active α-CGRP state. Electrophysiological recordings revealed decreases in abnormal thalamic firing during the stimulation-on condition.

**Conclusion:**

Our findings suggest that optical motor cortex stimulation can alleviate pain behaviors in a rat model of trigeminal neuropathic pain. Transmission of trigeminal pain signals can be modulated via knock-down of α-CGRP and optical motor cortex stimulation.

## Introduction

Trigeminal neuralgia (TN) is a facial pain syndrome characterized by paroxysmal pain attacks along the distribution of the trigeminal nerve. Among the two forms (typical and atypical) of TN, most typical cases of TN respond well to surgical treatments such as rhizotomy or, microvascular decompression. However, in case of patients with atypical TN (e.g., TN associated with disease or lesions in the trigeminal sensory system), the condition is often refractory to conventional treatment strategies [1, 2]. Therefore, finding effective alternative treatment approaches are indispensable.

In various chronic pain syndromes, the primary motor cortex (M1) plays a key role in the modulation of pain. Motor cortex stimulation (MCS) was first performed by Tsubokawa et al. in 1991 for the treatment of central pain, yielding promising results. MCS has since been for the treatment of several intractable pain syndromes, including, post-stroke pain, phantom limb pain, pain associated with multiple sclerosis/spinal cord injury pain, and postherpetic neuralgia. Studies have reported that > 75% of patients with TN achieve good or excellent pain relief following MCS [3–6]. However, both tolerance to MCS and the clinical efficacy of treatment appear to wane over time, ultimately resulting in short-term outcomes [1, 7]. Hence, further studies are required to determine the true benefits and risks of MCS and to find potential measures which can act as an adjuvant for maintaining a sustainable long-term result after MCS in patients with trigeminal neuropathic pain.

To understand the neural substrates of behavior, functional dissection of neural circuits is crucial. Various approaches have been used to intervene with neural dynamism but via the delivery of specific wavelengths of light following the introduction of genes that encode light-sensitive transmembrane channels, the Optogenetics and it’s advancement of strategies has enabled the precise temporal and spatial control of specific neural populations [8, 9]. Several animal studies have reported significant interactions among different brain areas in chronic pain, as well as the effects of therapeutic optogenetics on such interactions. Accumulating evidence has demonstrated that optogenetic stimulation of the brain exerts an impact on both physical and emotional aspects of pain, as it provides better temporal specificity than pharmacologic or electrical interventions [10–12]. Although the precise mechanism underlying its effects remains to be fully elucidated, optogenetic motor cortex stimulation may represent an alternative for patients in whom conventional treatment strategies such as pharmacological or, deep brain stimulation (DBS) have proven to fail to deliver satisfactory outcomes.

Calcitonin gene-related peptide (CGRP) is considered the most important neuropeptide in the trigeminal system, exhibiting an ascendant presence in TRPV1-expressing sensory neurons as α-CGRP. Studies have revealed that α-CGRP plays a key role in the trigeminal nociceptive system, and antibodies directed towards CGRP and CGRP receptor antagonists showing promising effects. Such studies suggest that α-CGRP plays a vital role in regulating pain in patients with chronic TN [13, 14].

Although effective in many cases, electrical MCS is not without risks and setbacks, necessitating further investigation of alternative treatment strategies such as optical MCS. Thus, in this present study, we utilized a rat model of TN to investigate behavioral changes following optogenetic modulation of neurons in cortical layer V of M1 in animals with inhibited or active state of α-CGRP.

## Materials and methods

### Experimental animal and ethical review

**Fig. 1:**
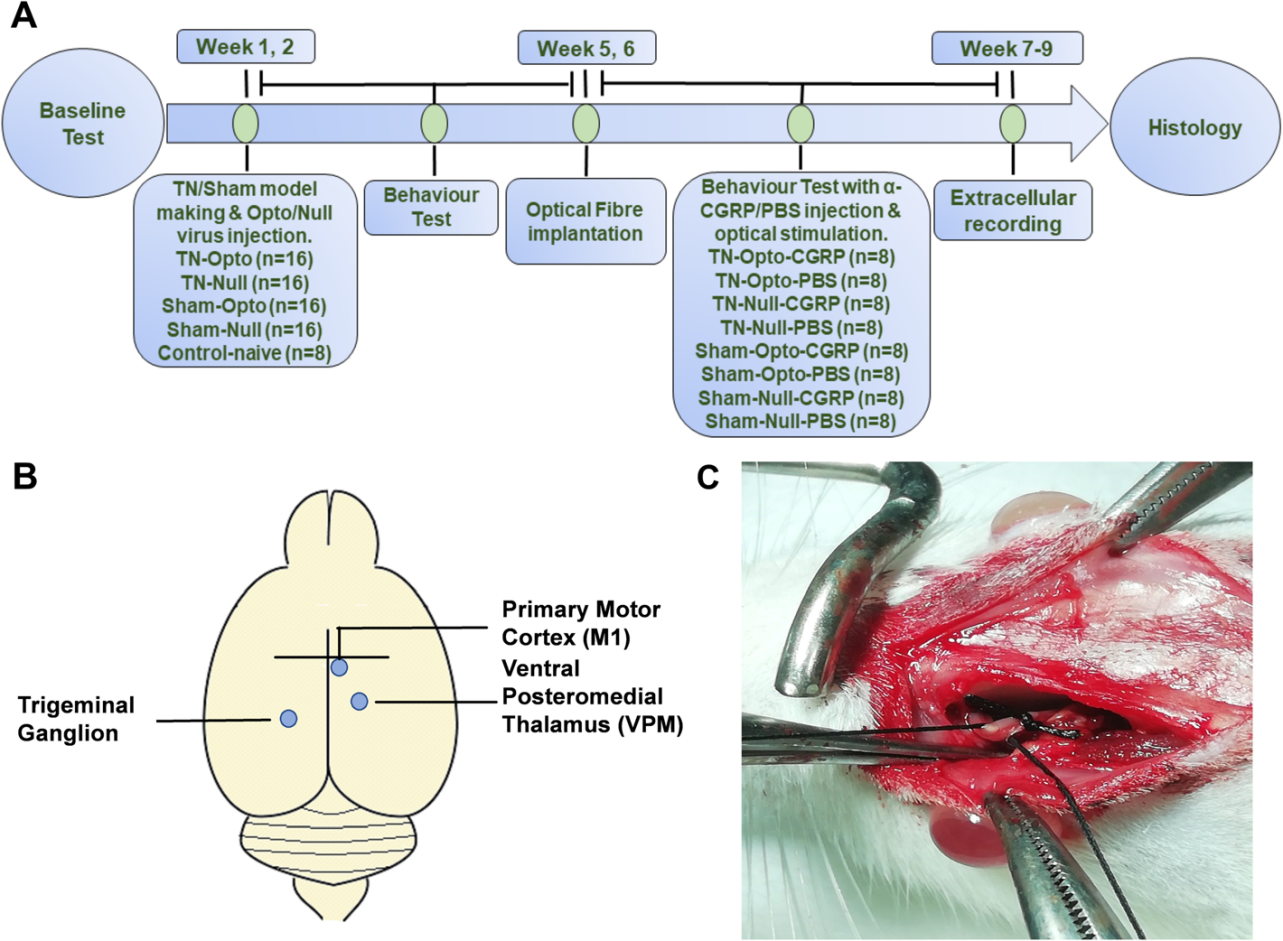
Experimental animal model and timeline. **a** Experimental timeline. **b** Schematic diagram of the experimental animal model and optogenetic virus injection site. C. Ligation of the ION to produce the TN model. ION: infraorbital nerve, TN: trigeminal neuralgia

The present study included 72 adult female Sprague-Dawley rats (age: 8 weeks; Koatech, Pyeongtaek, Korea), each weighing 200–250 g on arrival. Animals were allowed ad libitum access to fresh food and water and maintained under a 12-h light/dark cycle at a humidity of 50–60%. We used female animals because it has been well established that trigeminal nerve-associated disorders are more prevalent among females than among males, and that CGRP expression is significantly higher in females than in males [13]. All animal tests were conducted in a randomized, double-blind, controlled manner. The animals were randomly divided into a TN group (*n* = 32), a sham group (*n* = 32) and a naive control group (*n* = 8). The timeline of the experimental protocol is shown in Fig. 1. By the Institutional Animal Care and Use Committee (IACUC) of Chungbuk National University (CBNUA-1346-20-02), all experiments were approved and all animal experiments were performed during the light period at the Laboratory Animal Research Center of Chungbuk National University.

### Generation of trigeminal neuralgia model

Ligation of the infraorbital nerve (ION) was performed in 32 animals in accordance with the procedure described by Melanie et al.*,* 2008 [15]. Animals were anesthetized with an intraperitoneal (i.p.) injection of a mixture of 15 mg/kg Zoletil (Zoletil50®, Virbac Laboratories, Carros, France) and 9 mg/kg Rompun (Rompun®, Bayer, Seoul, South Korea) in saline, following which they were mounted onto the surgical field in a prone position. The skin above the eye was then shaved, and the animals were placed in a stereotaxic frame. Ophthalmic ointment was applied to the cornea to prevent damage associated with drying of the eyes. A skin incision of approximately 7 mm in length was made along the curve of the frontal bone in the anterior-posterior direction, 2 mm above the left eye. Moving laterally, the fascia and muscle were gently separated from the bone using a periosteal elevator. The ION could be observed on the maxillary bone following retraction of the eye. After revealing the ION, we prepared for ligature placement by gently freeing approximately 8 mm of the ION from the surrounding connective tissue. The ION was stretched slightly using a blunt needle with a curved head for ligature placement. The two ligatures were gently placed 3–4 mm apart, following which they were tightened until the ION was barely constricted. Finally, the incision above the eye was sutured with silk (3–0). Sham animals (*n* = 32) underwent identical procedures, with the exception of ligature placement. After surgery, fresh pelleted food and water were given to the rats ad libitum and checked for survival every day for at least 1 week. Naive animals did not go through above described surgical procedure.

### Injection of optogenetic viral vector

Animals of the TN group were randomly divided into two groups. Sixteen rats were subjected to optogenetic viral vector (AAV2-CaMKII-hChR2-EYFP) injection contralateral to the ION, while the remaining 16 were subjected to null virus (AAV2- CaMKII-EYFP) injection. Vectors were intracranially injected into layer V of M1 (AP − 1 mm, ML 1.5 mm, DV − 1.5 mm [16–18];) under general anesthesia. Animals of the sham group were also divided into two groups for injection of either the optogenetic virus or null virus. We injected an adeno-associated virus carrying the ChR2-EYFP fusion protein under the control of an excitatory neuron-specific CaMKII promoter. Prior to virus injection, anesthesia procedure was performed via an intraperitoneal injection of a mixture of 15 mg/kg tiletamine/zolazepam (Zoletil50®, Virbac Laboratories, Carros, France) and 9 mg/kg xylazine (Rompun®, Bayer, Seoul, Seoul, South Korea). Then, 2 μl of virus was injected using a Hamilton syringe and an automatic micro-syringe pump at a rate of 0.3 μl/min. Then the needle was kept in the same place after injection for 5 min to ensure virus absorption, following which it was slowly retracted.

### Behavioral testing

All behavior tests were conducted 1 day before (baseline) and every 7 days after model making. The behavior tests were performed in the following sequence:

#### Air-puff test

Animals were placed in a Plexiglas cage (20 × 20 × 14 cm) with a grid floor under quiet condition. Rats were kept in the room for at least 30 min to get habituated to the environment. Continuous puffs of air (starting from 5 psi (pound per square inch), duration: 4 s) were delivered to the ipsilateral side to the ION of the face at intervals of 10 s. Air puffs were delivered through a narrow tubing tip placed approximately 1 cm from the face at an angle of 90°. The psi was increased over trials and during administration of the air puff, we measured and evaluated the psi level of air puff at which aggressive behavioral responses such as biting or turning the head were exhibited by the animal. If the animal exhibited no response to a stimulus of at least 40 psi, the test was ended, and the animal was given a break for a few minutes. Mechanical thresholds were assessed and illustrated > 50% of the overall responses [2].

#### Mechanical allodynia test

Orofacial sensitivity to mechanical stimulation was assessed using von Frey filaments. Animals were first acclimated in a Plexiglas cage for 1 h. During this time, von Frey filaments were brought near the animals every 5 min for habituation. Animals were allowed to explore the filaments for a few seconds before they were removed. Stimuli were administered after the animals had become accustomed to the filament when placed near the center of the vibrissal pad within the territory of ipsilateral ION. The intensity of stimulation was gradually increased. The lowest force (in grams) required to elicit reactions such as immediate withdrawal, attacking the filament by biting or grabbling, escape behaviors, or asymmetric stroking of the face was recorded as the response threshold force [19].

#### Facial cold hyperalgesia test

Animals were placed in the same Plexiglas cage described above. For this test, a few drops of acetone were placed on the vibrissal pad ipsilateral to the ION using a glass syringe, following which we counted the number of scratching/rubbing behaviors over the next 2 min. Body parts other than the face were excluded for this assessment [2].

### Optic fiber implantation

Four weeks after optogenetic virus inoculation, animals were positioned in a stereotactic frame following induction of anesthesia. An optic fiber was implanted into the skull contralateral to the ION (AP: − 1 mm, ML: 1.5 mm) for the transmission of laser pulses to layer V of M1. Optic fibers (200 μm core, 230 μm outer diameter, numerical aperture of 0.48, hard polymer cladding type, Doric Lenses; Québec City, Québec, Canada) were cut to a length of 1.4 mm to optimize targeting of M1. Dental cement was used to fix the fiber firmly in place (Ortho-ject Pound Package, Lang Dental, USA).

### Selective knock-down of α-CGRP in trigeminal ganglion

Before performing the behavioral tests and extracellular recording with optic stimulation, animals in each group (TN-Opto, TN-Null, Sham-Opto, and Sham-Null) were again divided into two sub-groups of 8 animals each. Animals in one group received an injection of α-CGRP (α-CGRP (8-37) (mouse, rat) trifluoroacetate salt, 1 mg/ml, BACHEM) as a CGRP receptor antagonist, while animals in the other group received an injection of PBS. Animals of CGRP group received an intravenous injection of 10 μl of α-CGRP 10 min prior to the behavior test. In case of extracellular recording, α-CGRP was injected directly into the ipsilateral trigeminal ganglion (AP: − 3.5 mm, ML: − 3.6 mm, DV: − 12 mm) [20] and the recording was performed after that. α-CGRP was injected using a Hamilton syringe and an automatic micro-syringe pump at a rate of 0.3 μl/min. After injection, the needle was kept in the same place for 5 min to ensure absorption of CGRP, following which it was slowly retracted.

### Optical stimulation

**Fig. 2:**
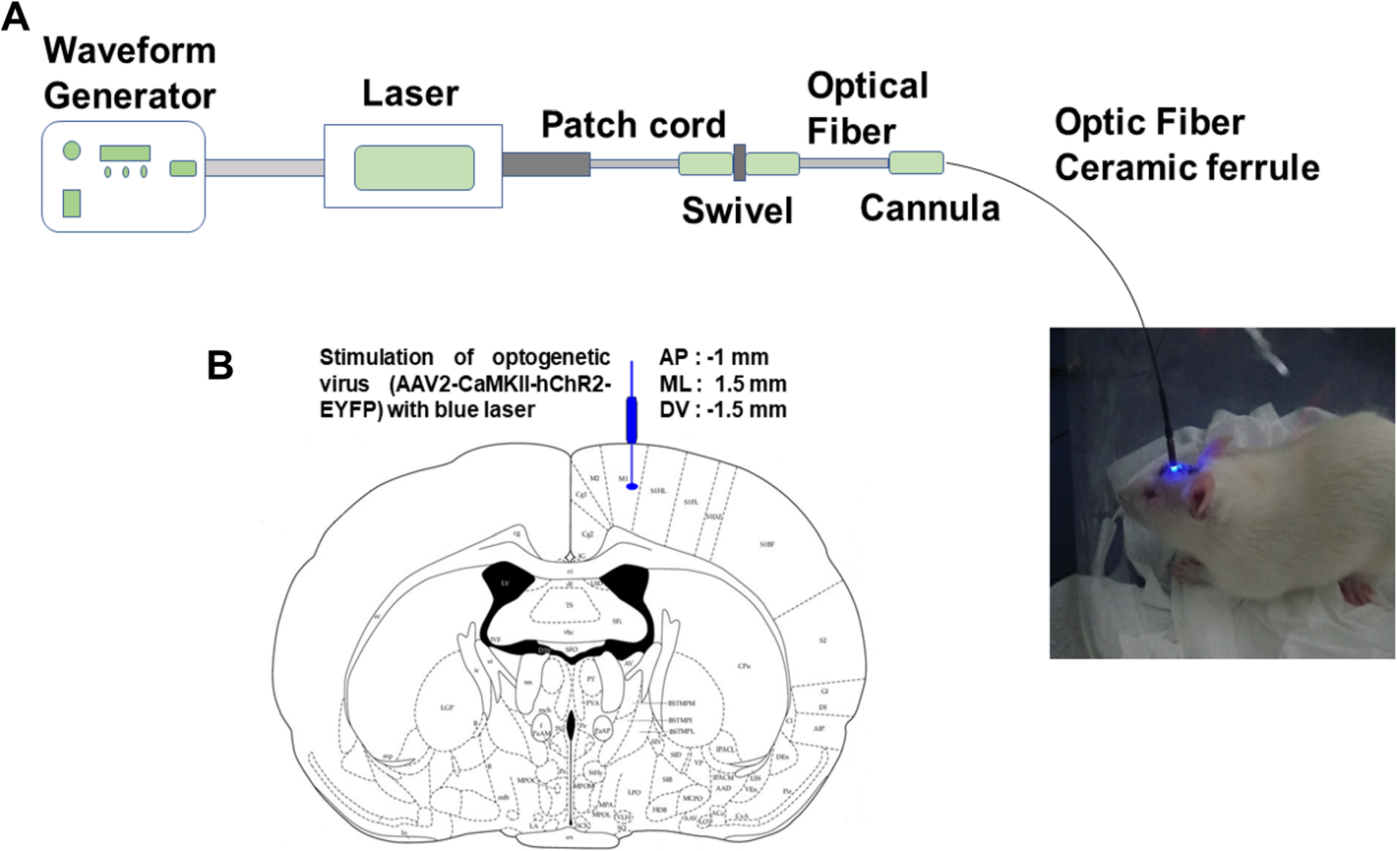
Optogenetic stimulation procedure. **a** Complete setup of optic stimulation. **b** Optic fiber implantation. **c** Stimulation of primary motor cortex using a blue laser

We used a laser power supply with a wavelength of 473 nm (ADR-700D, Shanghai, China) and a waveform generator (Keysight 33511b-CFG001, Keysight, Santa Rosa, CA, USA) to regulate the waveform and pulse width of the laser (Fig. 2). The laser’s intensity was set to 10 mW, the pulse width was set to 4 ms, the pulses were set to 20 Hz and the duration of stimulation was 5 min [21]. Behavioral differences were examined in animals inoculated with the optogenetic virus (pre, stim, post) to determine the effects of optical neuromodulation.

### In vivo extracellular recording

Six weeks after ligation of the ION, rats were anesthetized with 15 mg/kg tiletamine/zolazepam and 9 mg/kg xylazine to prepare for extracellular recordings. Extracellular recordings were obtained from the VPM (AP − 3.5 mm, ML 2.8 mm, DV − 6 mm) [2] using a single electrode. Following a 20-min resting condition, thalamic neuronal spikes and firing rate were recorded during the pre-stimulation, stimulation (blue: 473 nm), and post-stimulation states in vivo. A glass-insulated carbon fiber microelectrode (Cat. No.: E1011–20, Carbostar-1, Kation Scientific, LLC, MN 55414 USA) was used for recording in the thalamus. Recordings were obtained for 5 min during each stage, with a 5-min gap between stages. We picked well-isolated clusters and recording of neuronal signals was performed using a Digital Lynx SX (Neuralynx, Bozeman, USA) data-acquisition system along with Cheetah software. We digitized and bandpass filtered at 40 kHz and 1 Hz - 5 kHz, respectively. We sorted offline using Spike Sorter 3D (Neuralynx Inc., Montana, USA). Neuronal discharge was evaluated in the contralateral VPM of TN model and sham animals. Analysis of the rate histograms (spikes/s) of lesioned animals under different optical conditions was accomplished using Neuroexplorer (Neuralynx Inc., Montana, USA).

### Histological examinations

The rats were deeply anesthetized and transcardially perfused with PBS followed by 4% paraformaldehyde. The brains and trigeminal ganglion were extracted and fixed overnight in the same post fixed solution, followed which they were dehydrated in 30% sucrose solution.

We embedded brains and trigeminal ganglions in optimal cutting temperature (O.C.T.) compound (Tissue Tek®- Sakura, USA), following which they were cryopreserved with liquid nitrogen and isopentane at − 79 °C. A cryostat (Thermo Scientific, Waltham, MA, USA) was used to cut coronal sections of the brain (20 μm) and trigeminal ganglion (10 μm). The brain sections were incubated with DAPI and mounted with coverslips for examination under fluorescence microscope. To observe the action of α-CGRP receptor antagonist in trigeminal ganglion, the animals were sacrificed immediately after the extracellular recording and the trigeminal ganglion was collected. Trigeminal ganglion sections were immunostained with anti-CGRP antibody (1:200, ab36001, Abcam), following which they were incubated in serum block solution for 1 h and with anti-αCGRP antibody overnight. The corresponding secondary antibody was applied prior to staining with DAB (Vector Laboratory, California, USA). Nuclei were counterstained with hematoxylin. Finally, the sections were dehydrated and mounted with coverslips and examined under a microscope.

### Analysis of the bursting and firing rates

Activity in thalamic neurons was expressed as overall firing rates (spikes/s) in each optical stimulation condition using NeuroExplorer software (Neuralynx Inc.). Activity was assessed for 5 min in each state: pre-stimulation, stimulation, and post-stimulation. We defined burst rates as a group of at least three spikes with a maximum interval of 4 ms between spikes, with an interval of 100 ms between bursts. We selected similar inter-spike interval histograms for comparison among the groups.

### Statistical analysis

Data were analyzed using GraphPad Prism (GraphPad Software version 8.4.2, Inc., San Diego, CA, USA) and represented as the mean ± standard deviation (SD). We performed either an unpaired *t*-test, two-way analysis of variance (ANOVA) with Tukey’s post hoc test, or a repeated-measures ANOVA depending on the conditions of the experiment. Behavioral tests were assessed based on the mean values for each of the three optical states. Unpaired *t*-tests were used to compare firing rates between TN and sham-operated animals. All the statistical data were measured as significant at *p* < 0.005.

## Results

### Changes in pain behaviors following ION ligation

**Fig. 3:**
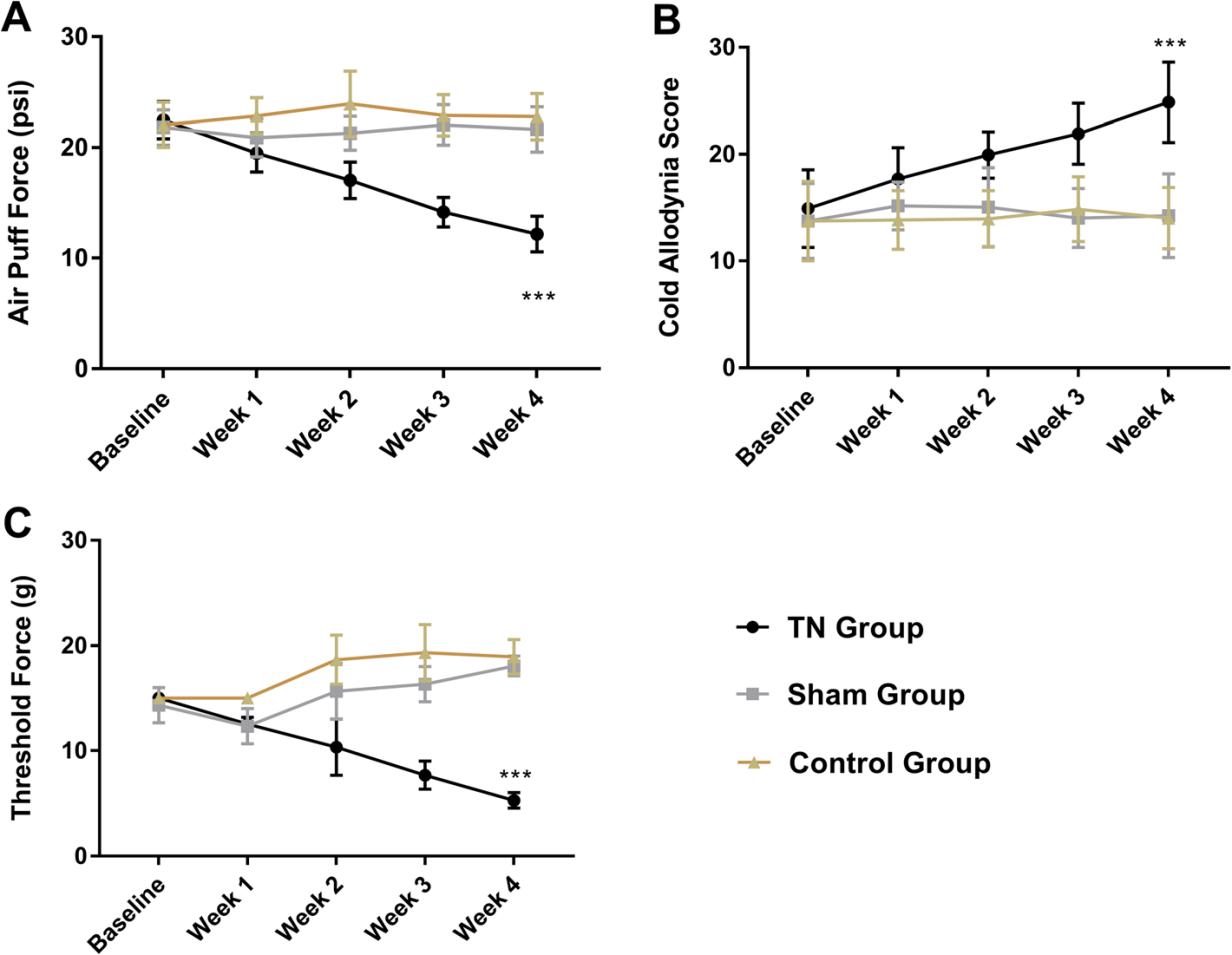
Behavioral test results for the TN, sham, and control groups. **a** Air-puff test results for the ipsilateral trigeminal facial area. **b** Cold hyperalgesia results for the ipsilateral facial area following treatment with acetone drops. **c** Mechanical allodynia (von Frey test) results for the ipsilateral facial side. ***, *p* < 0.001, significant difference determined via an analysis of variance (ANOVA)

Following ION ligation, behavioral test scores suggested the presence of chronic pain in TN animals, whereas no significant changes in behavioral scores were observed in sham and control animals. In TN group, a two-way ANOVA revealed significant, gradual decreases in air-puff test scores over the 4 weeks (22.46 ± 1.68 psi to 12.17 ± 1.62 psi; *F* (2, 165) = 180.1, *p* < 0.001; Fig. 3a). In addition, facial cold hyperalgesia scores significantly increased from 14.90 ± 1.64 to 24.85 ± 1.76 (two-way ANOVA, *F* (2, 165) = 48.59, *p* < 0.001; Fig. 3b). Mechanical allodynia scores also significantly decreased from 15 ± 00 g to 5.27 ± 0.73 g following surgery (*F* (2,165) = 259.9, *p* < 0.001; Fig. 3c).

### Attenuation of hyperalgesia via α-CGRP injection and optogenetic stimulation

**Fig. 4:**
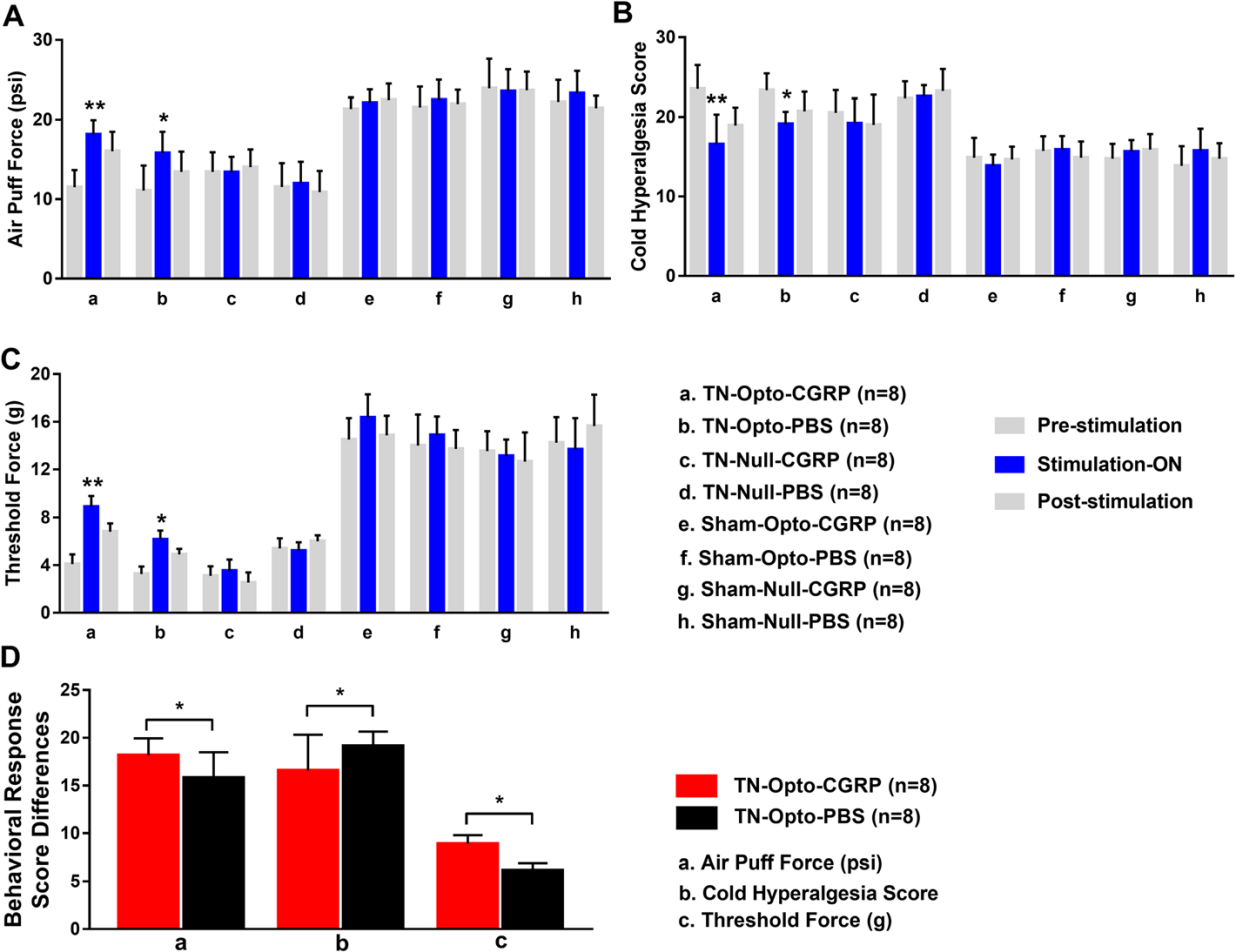
Changes in behavioral responses following blue laser stimulation (**a**-**c**) along with knocking down of α-CGRP (**d**). Results for the air-puff test (**a**), cold hyperalgesia test (**b**), and von Frey test (**c**) for all eight animal groups. Only the TN-Opto-CGRP (**a**) and TN-Opto-PBS groups (**b**) exhibited significant changes in behavioral scores during the stimulation ON condition. No significant changes in behavior were observed in other animal groups during the stimulation ON condition. **d** Animals with α-CGRP injection exhibited more improvement in behavioral tests than animals with PBS injection. **a** Air puff force differences, **b** Cold hyperalgesia score differences and (**c**) Mechanical allodynia score differences between TN-Opto-CGRP and TN-Opto-PBS group. *, *p* < 0.05; **, *p* < 0.01, significant difference determined via an analysis of variance (ANOVA)

Optical modulation induced significant changes in behavioral scores in the TN group. Our findings indicated that optogenetic stimulation resulted in neuromodulation of cells in layer V of M1. TN-Opto-CGRP animals exhibited significant alterations of behavioral responses in air-puff test during the stimulation-ON condition. For these animals, a two-way ANOVA revealed a significant effect of optogenetic modulation (pre-stimulation: 11.523 ± 2.13 psi; stimulation ON: 18.16 ± 1.78 psi; post-stimulation: 16.045 ± 2.44 psi; *F* (2, 24) = 36.65, *p* < 0.01) (Fig. 4A(a)). Significant decreases in cold hyperalgesia were also observed during the ON condition (pre-stimulation: 23.5243 ± 2.9854; stimulation ON: 16.563 ± 3.76; post-stimulation: 18.943 ± 2.2476; two-way ANOVA, *F* (2, 24) = 39.55, *p* < 0.01) (Fig. 4B(a)). Mechanical allodynia scores also improved in TN-Opto-CGRP animals during the ON condition (pre-stimulation: 4.1 ± 0.8 g; stimulation ON: 8.9 ± 0.9 g; post-stimulation: 6.8 ± 0.7 g; two-way ANOVA, *F* (2, 24) = 24.21, *p* < 0.05) (Fig. 4C(a)).

TN-Opto-PBS animals also exhibited significant changes in behavioral responses due to optogenetic stimulation, including increases in air-puff test scores (pre-stimulation: 11.10 ± 3.13 psi; stimulation ON: 15.81 ± 2.68 psi; post-stimulation: 13.45 ± 2.54 psi; two-way ANOVA, *F* (2, 24) = 22.673, *p* < 0.05) (Fig. 4A(b)). These animals also exhibited significant decreases in cold hyperalgesia during the ON condition (pre-stimulation: 23.345 ± 2.11; stimulation ON: 19.13 ± 1.53; post-stimulation: 20.711 ± 2.46; two-way ANOVA, *F* (2, 24) = 25.88, *p* < 0.05) (Fig. 4B(b)). In addition, we observed improvements in mechanical allodynia scores in the TN-Opto-PBS group during the ON condition (pre-stimulation: 3.25 ± 0.64 g; stimulation ON: 6.15 ± 0.75 g; post-stimulation: 4.90 ± 0.48 g; two-way ANOVA, *F* (2, 24) 22.03, *p* < 0.05) (Fig. 4C(b)). The other animal groups (TN-Null-CGRP, TN-Null-PBS, Sham-Opto-CGRP, Sham-Opto-PBS, Sham-Null-CGRP, Sham-Null-PBS) exhibited no significant changes in behavior due to optogenetic modulation or null virus injection (Fig. 4A(c-h), B(c-h), C(c-h)).

We further evaluated the extent of improvement in animals by optic stimulation along with or without α-CGRP knock down and it was found that TN-Opto-CGRP grouped animals showed more improvement than TN-Opto-PBS group (Fig. 4d).

These findings support our hypothesis that stimulation of neurons in layer V of M1 exerts an antinociceptive effect in TN.

### In vivo extracellular recording data

**Fig. 5:**
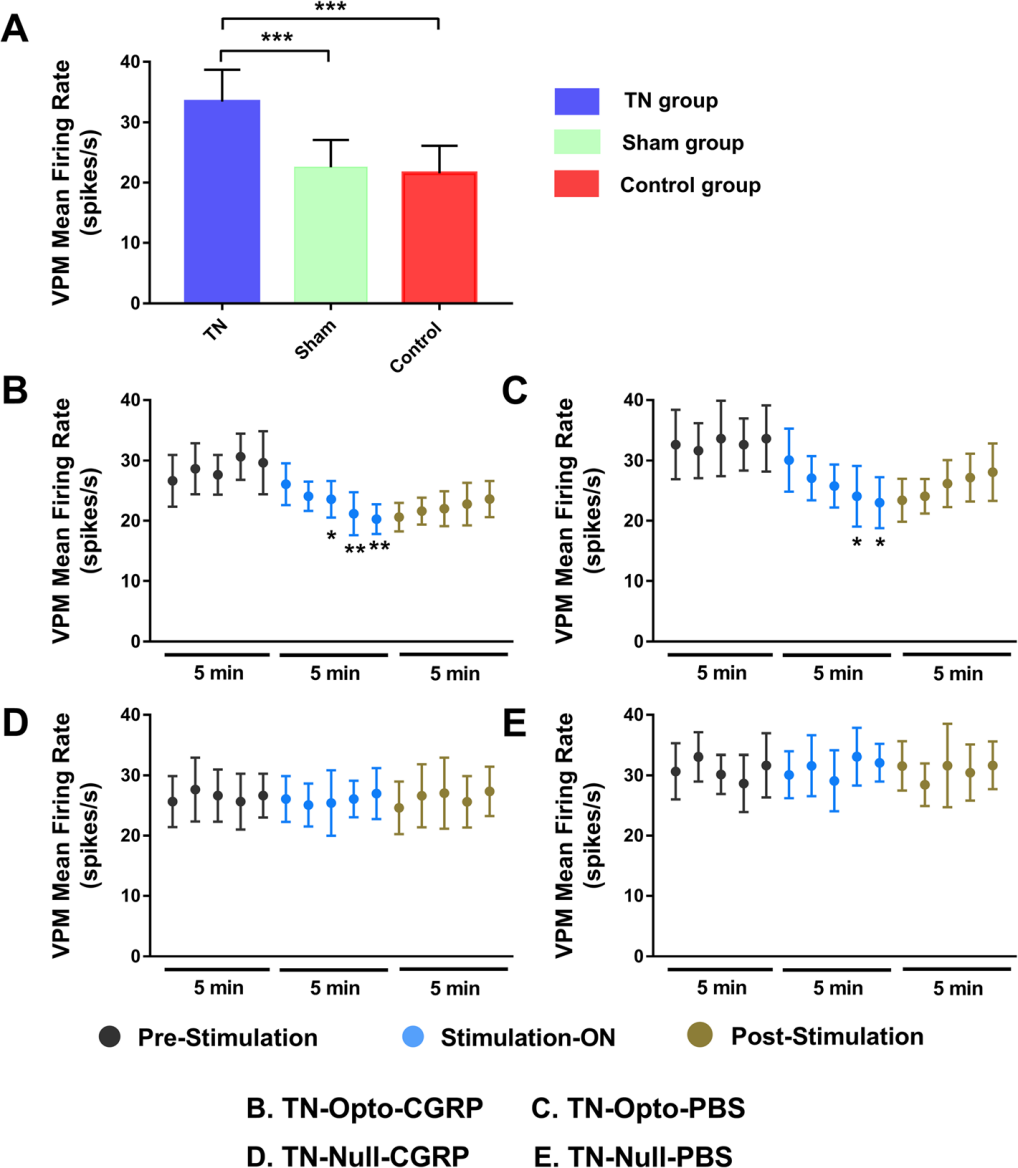
Electrophysiological findings for VPM thalamic output due to optogenetic stimulation of the motor cortex. **a** Evoked firing rates in VPM neurons in TN animals versus sham and control animals (***, *p* < 0.001, significant difference determined using unpaired *t*-tests). **b**, **c** In vivo recordings of the VPM thalamus from TN-Opto animals. **b** Findings for the TN-Opto-CGRP group. **c** Findings for the TN-Opto-PBS group. Decrease in firing output (spikes/s) were observed during the stimulation ON period. **d**, **e** No changes in firing rates of VPM thalamus were observed in the TN-Null-CGRP and TN-Null-PBS groups, respectively (two-way analysis of variance (ANOVA) *, *p* < 0.05; **, *p* < 0.01

To confirm whether changes in neuronal activity had occurred, we examined firing rates in VPM neurons in the TN, sham, and control groups. Significantly higher firing rates were observed in TN-lesioned rats (33.435 ± 5.233 spikes/s) than in sham rats (22.563 ± 4.534 spikes/s, unpaired *t-*test (t, df) = (8.882, 62), *p* < 0.001) or control rats (21.564 ± 4.563 spikes/s, unpaired *t-*test (t, df) = (5.87, 38), *p* < 0.001) (Fig. 5a).

We assessed the effects of optical stimulation on M1 in the TN-Opto and TN-Null groups using in vivo extracellular recordings. Optogenetic stimulation induced significant changes in VPM neuron activity in the TN-Opto group. Spike rates in the pre-stimulation, stimulation ON, and post-stimulation conditions in the TN-Opto-CGRP group were 27.66 ± 4.19, 20.85 ± 2.99, and 22.09 ± 2.73, respectively. A two-way ANOVA revealed significant effects of optic stimulation (*F* (2,345) = 59.09, *p* < 0.01) (Fig. 5b). Spike rates for the pre-stimulation, stimulation ON, and post-stimulation conditions in the TN-Opto-PBS group were 32.80 ± 5.28, 26.45 ± 4.35, and 28.03 ± 3.33, respectively (*F* (2,345) = 37.53, *p* < 0.05) (Fig. 5c). No significant changes in thalamic firing rates were observed in either the TN-Null-CGRP or TN-Null-PBS groups (Fig. 5d and e).

**Fig. 6:**
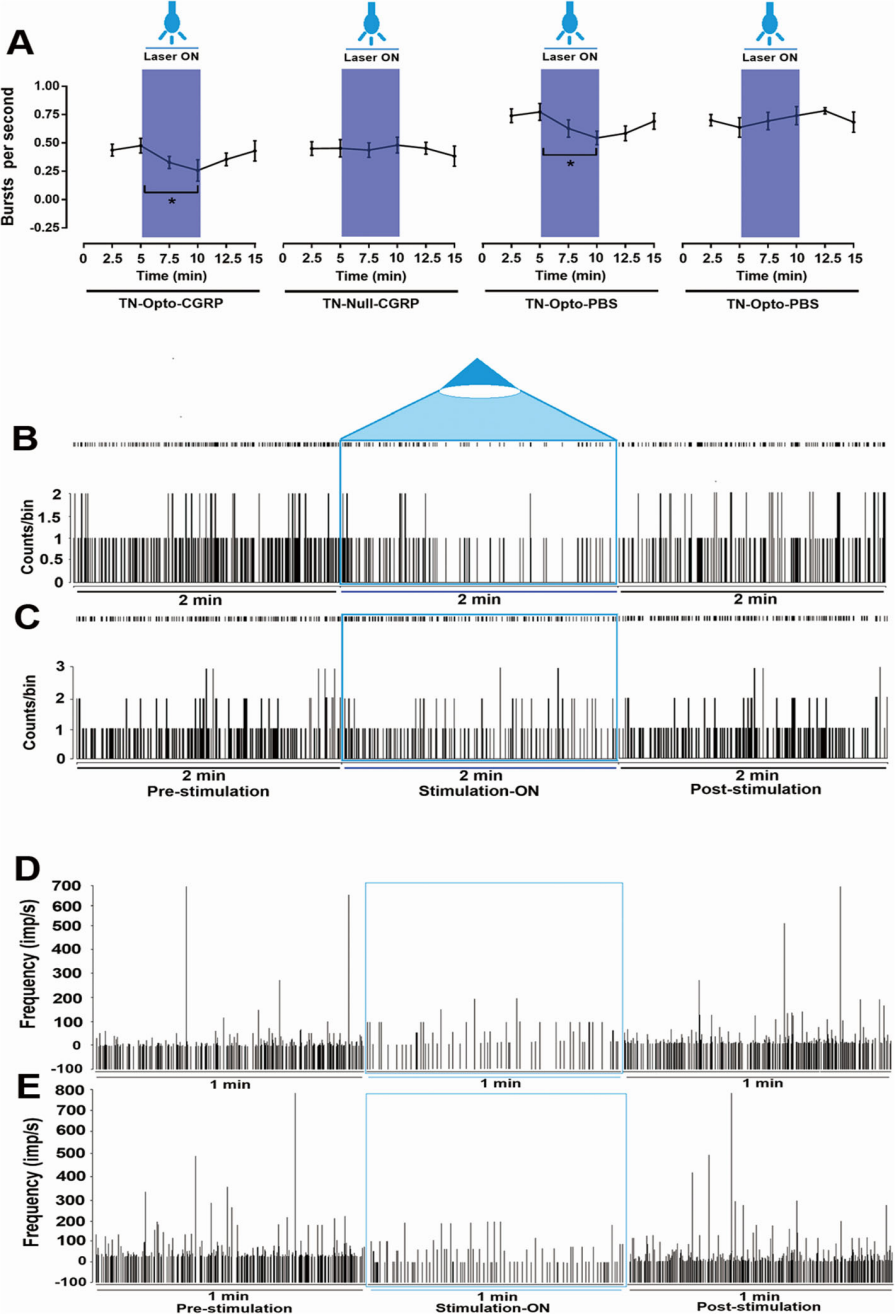
Burst firing rates in TN animals following optogenetic stimulation (**a**). Significant changes were observed in the TN-Opto-CGRP and TN-Opto-PBS groups. *, *p* < 0.05; **, analysis of variance (ANOVA). **b**, **c** Peri-event raster histogram of VPM neuron responses in the TN-Opto-CGRP and TN-Opto-PBS groups, respectively. VPM firing rates decreases during optical stimulation. Bin size = 50 ms. **d**, **e** Instantaneous firing frequency for VPM neurons in TN-Opto-CGRP and TN-Opto-PBS rats, respectively

Burst rates appeared to decrease during optogenetic stimulation in the TN-Opto group. Thalamic burst rates for the pre-stimulation, stimulation ON, and post-stimulation conditions in the TN-Opto-CGRP group were 0.44 ± 0.08/s, 0.28 ± 0.06/s, and 0.40 ± 0.10/s. respectively. A two-way ANOVA revealed significant effects of optogenetic stimulation (*F* (2,276) = 5.45, *p* < 0.05) (Fig. 6a). Optogenetic stimulation also significantly decreased burst rates in the TN-Opto-PBS group. Thalamic burst rates for the pre-stimulation, stimulation ON, and post-stimulation conditions in the TN-Opto-PBS group were 0.75 ± 0.07/s, 0.58 ± 0.06/s, 0.67 ± 0.10/s, respectively. A two-way ANOVA revealed significant effects of optogenetic stimulation (*F* (2,276) = 3.57, *p* < 0.05) (Fig. 6a). However, stimulation induced no significant changes in burst rates in the TN-Null-CGRP and TN-Null-PBS groups. In both the TN-Opto-CGRP and TN-Opto-PBS groups, we observed greater inhibition of thalamic responses during the stimulation ON state than during the stimulation OFF state (Fig. 6b and c). Instantaneous firing frequencies also decreased during the stimulation ON condition (Fig. 6d and e).

These results suggest that optogenetic stimulation of M1 can attenuate thalamic discharge in TN rats. Optogenetic MCS showed significant improvement in pain response, though along with knock down of α-CGRP it provided more improved and long-term pain relief. No effects of laser stimulation on thalamic firing patterns were observed in the TN-Null-CGRP and TN-Null-PBS grouped animals.

### Viral expression in motor cortex and inactivation of α-CGRP in trigeminal ganglion

**Fig. 7:**
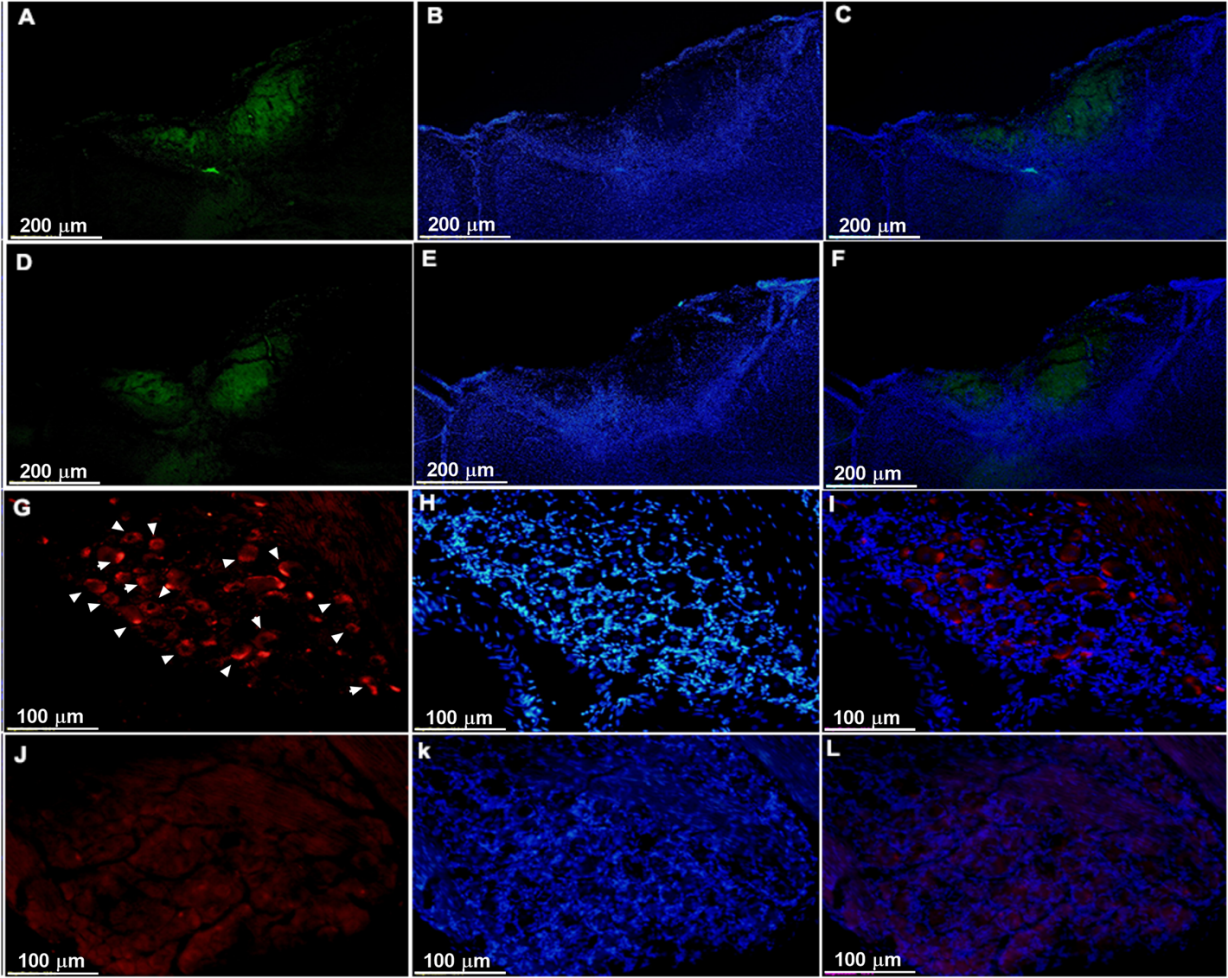
Immunofluorescence results for viral expression in the primary motor cortex (**a**-**f**) and CGRP neuron expression in the trigeminal ganglion (**g**-**l**). **a**, **b**, **c** Optogenetic viral expression in the motor cortex of TN-Opto animals. **d**, **e**, **f** Null viral expression in the motor cortex of TN-Null animals. **a**, **d** EYFP, **b**, **e** DAPI, and **c**, **f** Merge. Scale bar = 200 μm. **g**-**l** PBS and α-CGRP-injected and DAPI-stained trigeminal ganglion cells. **g-i** Presence of anti-CGRP antibody binding due to active state of α-CGRP neuron (arrow) within the trigeminal ganglion of PBS injected animal (**j**-**l**). α-CGRP injected and DAPI-stained trigeminal ganglion cells showing absence of CGRP neurons due to inhibition of α-CGRP neurons within the trigeminal ganglion. **g**, **j** CGRP, **h**, **k** DAPI, and **i**, **l** Merge. Scale bar = 100 μm

The optogenetic target for the present study was layer V in M1, which was stereotaxically located using a rat atlas. We observed expression of the optogenetic and null viruses in the contralateral M1 using immunofluorescence (Fig. 7a-f). By using a fluorescence microscope and ImageJ software, viral expression in neurons stained using EYFP and DAPI were obtained. We observed the presence of anti-CGRP antibody binding in trigeminal ganglion cells in animals injected with PBS and anti-CGRP binding was absent in α-CGRP injected animals (Fig. 7g-l).

## Discussion

In the present study, we aimed to investigate whether optical stimulation of contralateral M1 can modulate chronic neuropathic pain in rats with ION injury. Here we presented results showing that such stimulation significantly improved behavioral responses to pain and modulated thalamic output. Animals with TNP exhibited pain relief responses during MCS by optogenetic system in both with and without α-CGRP knockdown condition. In vivo extracellular recording results proved decreased thalamic firing rates while TN animals were subjected to optogenetic stimulation. All these findings provide foundation for the effectiveness of optogenetic MCS in the treatment of TNP.

TN is the most common form of cranial neuralgia. CCI-ION is a common method of mimicking TN neuropathic pain in animal models. Onset of TN can be triggered by certain factors such as nerve compression by intracranial and extra cranial tumors or vascular anomalies, demyelinating conditions, nerve trauma, or infectious agents. These conditions can decrease the excitability threshold of affected fibers, thereby promoting improper propagation towards surrounding fibers. Trigeminal afferents project to the mesencephalic nucleus, the principle sensory nucleus of the trigeminal nerve, and the spinal nucleus of the trigeminal nerve by means of the trigeminal ganglion. While non-painful sensory input projects to the principle sensory nucleus, nociceptive information travels down to the spinal nucleus of the trigeminal nerve. These mutually exclusive sensory signals are integrated in the sensory area of the thalamus (i.e., the VPM). In patients with TN, aberrant sensory information leads to the transmission of inappropriate sensory signals to the thalamus, initiating the sensation of pain and changes in behavior [22–27], as observed in our study that after making CCI-ION rat model, animals showed painful behavioral responses and behavior test’s scores were significantly different from sham and control grouped animals.

The function of the trigeminal system is regulated by numerous factors including neuropeptides. CGRP is believed to be a leading contributor to the neurogenic inflammation underlying various disorders involving craniofacial structures. Research has indicated that α-CGRP neurons are necessary for neuropathic hypersensitivity to mechanical, cold, and heat stimuli; spontaneous pain; and sensitization of mechanical currents in afferent somata. The release of CGRP and other excitatory neurotransmitters from TG neurons in the trigeminal nucleus caudalis (TNC) can excite second-order neurons in the TNC, directly contributing to central sensitization, allodynia, and hyperalgesia. Thermal hyperalgesia is significantly inhibited in α-CGRP knockout mice. Furthermore, chemical ablation of α-CGRP neurons suggests that they are essential for the detection of cold and heat, and for heat hypersensitivity after both neuropathic and inflammatory injuries. Additional studies have reported that treatment with a CGRP antagonist decreases injury-induced mechanical hypersensitivity [13, 14, 28–31]. The findings of the present study are in accordance with previous observations of improvements as animal with α-CGRP inhibition following optic stimulation showed better improvement in hypersensitivity than animals treated with optical stimulation alone.

M1 exhibits wide connections to diverse areas of the brain and many spinal efferent and afferent fibers that modulate the transmission of signals associated with painful stimuli. Thus, stimulation of M1 can initiate changes in other brain areas. One advantage of MCS relative to other neurostimulation techniques such as DBS or SCS is that MCS-induced analgesia is not associated with paresthesias [32–34]. By activating the top-down controls associated with intracortical horizontal fibers, MCS exerts its effects which can elicit paresthesias via the activation of non-nociceptive third- or fourth-order somatosensory neurons within the thalamic nucleus [27, 35–37]. The motor cortex is composed of six layers, each playing a distinct role in cerebral function. Among them, layer II/III axons usually send excitatory inputs to pyramidal neurons in layers V and VI. Two classes of projection neurons are important for motor control: corticospinal and corticostriatal neurons. Excitation of layer V neurons due to MCS appears to trigger activation in the striatum, in turn causing rapid phasic alterations in the thalamus by means of GABAergic neurons [38, 39]. Several PET and fMRI studies have indicated that MCS is accompanied by increased blood flow and activation of pain-related brain regions associated with endogenous opioid secretion. These regions lie adjacent to outer brain areas (e.g., orbitofrontal cortex) as well as remote inner brain structures including the insula; anterior, middle, and posterior cingulate cortices; putamen; thalamus; and portions of the brainstem (e.g., PAG and pons) [40–43]. Neuromodulation of M1 may also alter activation at NMDA/AMPA receptors and concentrations of GABA/glutamate at synapses in S1, S2, the thalamus, brainstem, ACC, prefrontal cortex, and other structures. Moreover, studies involving experimental models of neuropathic pain suggest that MCS-induced antinociceptive effects involve the rostroventromedial medulla as well as descending serotoninergic pathways [44–49].

In the present study, we utilized optogenetic techniques to stimulate M1 in order to induce circuit-specific neuromodulation of neuronal activity in layer V of M1 [10, 50, 51]. To regulate cellular activity with high spatiotemporal resolution, we relied on heterologous expression of the photosensitive actuator channelrhodopsin2 (ChR2), which enabled exact quantitative linking between optical excitation and activation of neurons [52–54]. Since ChR2 is genetically targetable, its expression is used as a powerful tool for increasing cytoplasmic Ca^2+^ concentrations or depolarizing the cell membrane [55, 56]. These channels open when activated by blue light (~ 473 nm) and were used to induce neuronal excitation in the present study [57, 58]. CaMKII is a glutamatergic, neuron-specific promoter that drives ChR2 expression. As a result, expression of ChR2 is specific to the excitatory CaMKII*α*-expressing cortical neuron population. As a result, the optical neural interface selectively activates excitatory neurons in the cortex. Our behavioral findings also support the notion that optogenetic MCS improves hypersensitivity by exerting antinociceptive effects as animals with optogenetic virus showed significant improvement than animals with null virus.

Our electrophysiological analyses revealed a robust effect of optical MCS on thalamic and mesencephalic neurons, which may explain the observed analgesic effects. The data we got from extracellular recording of TN-Opto-CGRP and TN-Opto-PBS grouped animals demonstrated significant changes of VPM thalamic responses. Among several structures, the thalamus receives pain signals from various pain pathways. Following peripheral nerve injury, changes in the rhythmic burst firing of thalamic neurons may contribute to the development of chronic pain. The somatotopic ordination of primary afferents found in each brainstem subnucleus is conserved by the trigemino-VPM pathway. The spinal trigeminal nucleus relays somatosensory orofacial information from the brainstem to the thalamus. Spino- and trigemino-thalamic fibers run through the caudo-rostral extent of the posterior triangular thalamic nucleus and then expand in the Po and VPM. The major trigeminal course to the somatosensory cortex is cognizant to project through the VPM and part of the Po. In accordance with our findings, several previous studies have reported electrophysiological hyperresponsiveness of the contralateral VPM in CCI-ION rats [2, 42, 47, 48, 59–63]. Optical stimulation of ChR2-transfected pyramidal neurons in layer V of M1 inhibits sensory inputs in the spinothalamic tract and modulates cortico-striatal neural circuitry, thereby reducing abnormal thalamic firing. Furthermore, thalamic burst rates during optogenetic stimulation also showed to be decreased in TN-Opto-CGRP and TN-Opto-PBS group.

There have been some limitations in our study. The post-operative brunt may have impact on chronic state of pain as we did not give any medications after surgical intervention. We did not examine the effects of other cortical structure’s stimulation. The use of anesthesia may have influenced rates of spontaneous thalamic discharge. We tried as much as possible to avoid the effect of stimulation with Von Frey filaments during neural recording. Furthermore, although we performed electrophysiological analyses of VPM activity in response to pain, we did not perform such analyses in the trigeminal ganglion.

Future studies could utilize the novel technique to determine different dopaminergic neuron’s distinct roles in OMCS-induced pain modulation. Apart from using glutamatergic neuron specific promoter to modulate M1, using of non-specific promoter or GABAergic specific promoter in OMCS may have different outcomes which requires further studies. As α-CGRP was used in our study, further studies with different neuropeptide or other molecules can be conducted to determine whether their infliction can facilitate gaining of desired outcomes by MCS. Precise temporal modulation of earmarked neuronal subtypes may guide to insights into clinically pertinent neuromodulation with less side effects and more sturdy therapeutic efficiency, moreover, insights into the cellular basis of systems-level neural circuit functioning responsible for chronic trigeminal neuropathic pain.

## Conclusion

Our findings indicated that optogenetic stimulation of M1 exerts an analgesic effect in a rat model of TN. Further improvements in pain were observed following treatment with α-CGRP, indicating that the central neuromodulation mechanism is independent of the control of peripheral pain. Optical activation of M1 via ChR2 may serve as an optical scalpel in TN. Targeted, temporally precise control of specific neural subtypes may lead to insights into clinically relevant neuromodulation with fewer side-effects and more robust therapeutic efficacy, as well as insights into the cellular basis of systems-level neural circuit function. Nonetheless, further studies are required in order to clarify the mechanisms by which optical MCS attenuates trigeminal neuropathic pain.

## Data Availability

The datasets used and/or analysed during the current study are available from the corresponding author on reasonable request.

## Abbreviations

TN: Trigeminal neuralgia
M1: Primary motor cortex
MCS: Motor cortex stimulation
DBS: Deep brain stimulation
CGRP: Calcitonin gene-related peptide
ION: Infraorbital nerve
VPM: Ventral Posteromedial nucleus of Thalamus
CCI-ION: Chronic constriction injury of ION
TNC: Trigeminal neural caudalis
